# Comparing outcomes of total hip arthroplasty between cirrhotic and non-cirrhotic patients through a propensity-matched analysis

**DOI:** 10.1007/s00402-026-06214-6

**Published:** 2026-03-09

**Authors:** Muhammad Haider, Farouk Khury, Jonathan Katzman, Patrick Connolly, Anzar Sarfraz, Ran Schwarzkopf, Claudette M. Lajam

**Affiliations:** 1 Department of Orthopedic Surgery, New York University Langone Medical Center, New York, USA; 2 The Ruth and Bruce Rappaport Faculty of Medicine, Technion Israel Institute of Technology, Haifa, Israel; 3 Division of Orthopedic Surgery, Rambam Health Care Campus, Haifa, Israel

**Keywords:** Cirrhosis, Total hip arthroplasty, Clinical outcomes, MELD, Peri-prosthetic joint

## Abstract

**Background:**

The impact of liver cirrhosis on surgical outcomes is well-known. This study aimed to compare postoperative outcomes of total hip arthroplasty (THA) in patients with versus without cirrhosis.

**Methods:**

A retrospective review was conducted of all patients who received a THA between 2012 and 2021 with a minimum of two years of clinical follow-up at a single, urban tertiary health center with lab results available to calculate Model for End-stage Liver Disease (MELD) scores. Using demographic variables, patients with and without cirrhosis underwent a 10:1 propensity score match. Short-term clinical outcomes were compared between cohorts. Cirrhotic patients were stratified based on their MELD score as mild (MELD < 10, *n* = 39) or moderate-to-severe (MELD ≥ 10, *n* = 10).

**Results:**

Of the 539 patients included in this study, 49 patients were in the cirrhotic group and 490 patients were in the non-cirrhotic group. Compared to non-cirrhotic and mild cirrhotic, moderate-to-severe cirrhotic THA patients had significantly higher incidence of 30-day (2.9% vs. 2.6% vs. 30.0%, *p* = 0.011) and 90-day readmissions (5.9% vs. 2.6% vs. 30.0%, *p* = 0.038) due to periprosthetic joint infection (PJI), and higher incidence of 90-day (3.1% vs. 2.6% vs. 20.0%, *p* = 0.024) and all-time revisions (1.4% vs. 5.1% vs. 20.0%, *p* = 0.016) due to PJI. There were no differences in overall 90-day reoperation (*p* = 0.115) and revision risk (*p* = 0.202) between non-cirrhotic, mild cirrhotic, and moderate-to-severe cirrhotic THA patients. Freedom from all-cause reoperations/revisions did not differ significantly (*p* = 0.479) between non-cirrhotic and cirrhotic THA patients at 120 months of follow-up.

**Conclusions:**

Cirrhotic patients, particularly those categorized as moderate-to-severe, undergoing THA may have higher risk of having a readmission or revision for PJI. However, overall 90-day readmission and revision risk were similar between non-cirrhotic and cirrhotic patients. Future research with larger sample sizes and databases is needed to further risk stratify, optimize and counsel cirrhosis patients surrounding THA.

## Introduction

As surgical techniques and perioperative care improves, THA is being offered to older and more medically complex patients [1]. It is important to evaluate the complication profile associated with THA in different patient populations in order to properly counsel these patients preoperatively on the risks and benefits of surgery. In addition, in the era of value-based care and bundled payment models for THA, understanding the potential increased cost of care for different patient cohorts undergoing elective surgery is highly relevant for surgeons, hospital systems, and insurance companies.

Cirrhosis is end-stage chronic liver disease as a result of alcoholism, viral hepatitis, or non-alcoholic fatty liver disease [2]. It is irreversible and can affect many different organ systems in the body [2]. Patients can develop impaired immune function, coagulopathy, nutritional deficiencies, encephalopathy and dementia as the disease progresses [2]. It is estimated that about 1% of the United States population has compensated cirrhosis, which may not present with any significant symptoms [3]. While many of these cases remain undiagnosed, the prevalence of *clinically documented* cirrhosis is rising among patients presenting for joint replacement, necessitating a clear understanding of perioperative risks.

There have been several large database studies that looked at outcomes in cirrhotic patients after THA [4–7]. These studies have found that cirrhotic patients are at an increased risk of complications and have an increased financial burden after elective THA [4–7]. Database studies are very powerful as they include large numbers of patients, but these studies rely on accurate diagnosis coding in the medical record. Overall, studies looking at outcomes in cirrhotic patients undergoing THA that define cirrhotic patients by MELD or ChildPugh scores, which are widely used and are a validated scoring systems, have been lacking [8, 9].

The purpose of this study is to evaluate the postoperative outcomes of THA in patients with and without cirrhosis as defined by MELD score.

## Methods

### Study design

A retrospective study was conducted at an urban, academic institution to investigate THA procedures in patients with cirrhosis. Institutional Review Board (IRB) approval was received prior to the initiation of the study. Patients were included if they underwent primary, elective THA due to primary or secondary osteoarthritis, femoral head avascular necrosis or adult hip dysplasia, and had complete electronic records and at least two years of follow-up visits detailing their postoperative progress as well as development of any complications, visits to the emergency department (ED), readmissions, reoperations or revisions. Patients were excluded from the study if they had any diagnosis of ongoing or previous soft tissue, bone, or joint infection, any ongoing immunosuppressant treatment, any active oncologic disease, any previous intervention performed on the same hip or knee joint, if the indication for surgery was not osteoarthritis, or if they had any missing data. Manual chart review was conducted to collect any missing data for cirrhotic patients. An electronic data warehouse (EDW) search identified 49 elective, primary THAs performed in patients with cirrhosis at the authors’ institution between April 1, 2012 and September 31, 2021 who had at least two years of follow-up. It is worth noting that it is a policy at our institution, that while patients with a Model for End-stage Liver Disease (MELD) score under ten are permitted to undergo surgery at our Orthopedic Specialty hospital, cirrhotic patients with a higher MELD score are referred to the main campus hospital for surgery, which is equipped with an intensive care unit and advanced medical services to support higher-risk patients. Importantly, this study includes data from all hospital sites, including both the specialty hospital and the main campus. While the location of surgery was determined by MELD score and clinical risk assessment, the surgical protocol was standardized and consistent across all sites. Therefore, differences in outcomes are not attributable to variations in surgical technique or perioperative management, but rather reflect patient-level factors. The inclusion of patients from both sites allows for a comprehensive analysis of cirrhotic patients across the MELD spectrum within a unified institutional framework.

### Data collection and outcome measures

Our institution’s electronic medical records (EMR) system (Epic Caboodle. Version 15; Verona, Wisconsin, USA) was utilized to collect baseline demographics, including sex, age, race, smoking status, American Society of Anesthesiologists (ASA) score, body mass index (BMI), and Charlson Comorbidity Index (CCI). Propensity-score matching (PSM) was based on age, sex, BMI, race, smoking status, ASA score, and CCI. PSM using the nearest neighbor method was performed using these demographic variables to create a similar cohort of THA recipients without cirrhosis. The 49 THA cases in patients with cirrhosis were ten-to-one propensity score matched to a cohort of 17,122 primary, elective THAs in patients without cirrhosis who had at least 2 years of clinical follow-up. The propensity scores were estimated using logistic regression, and matching was performed using the Nearest Neighbor method. Following matching, the standardized mean differences (SMD) for all covariates were below 0.25, indicating adequate balance between both groups. After the matched cohorts were created, chi-squared tests and independent samples *t*-tests validated that there were no significant demographic differences between the cirrhotic and non-cirrhotic matched cohorts. Lab results for all patients with cirrhosis were collected and MELD scores were calculated.

**Fig. 1:**
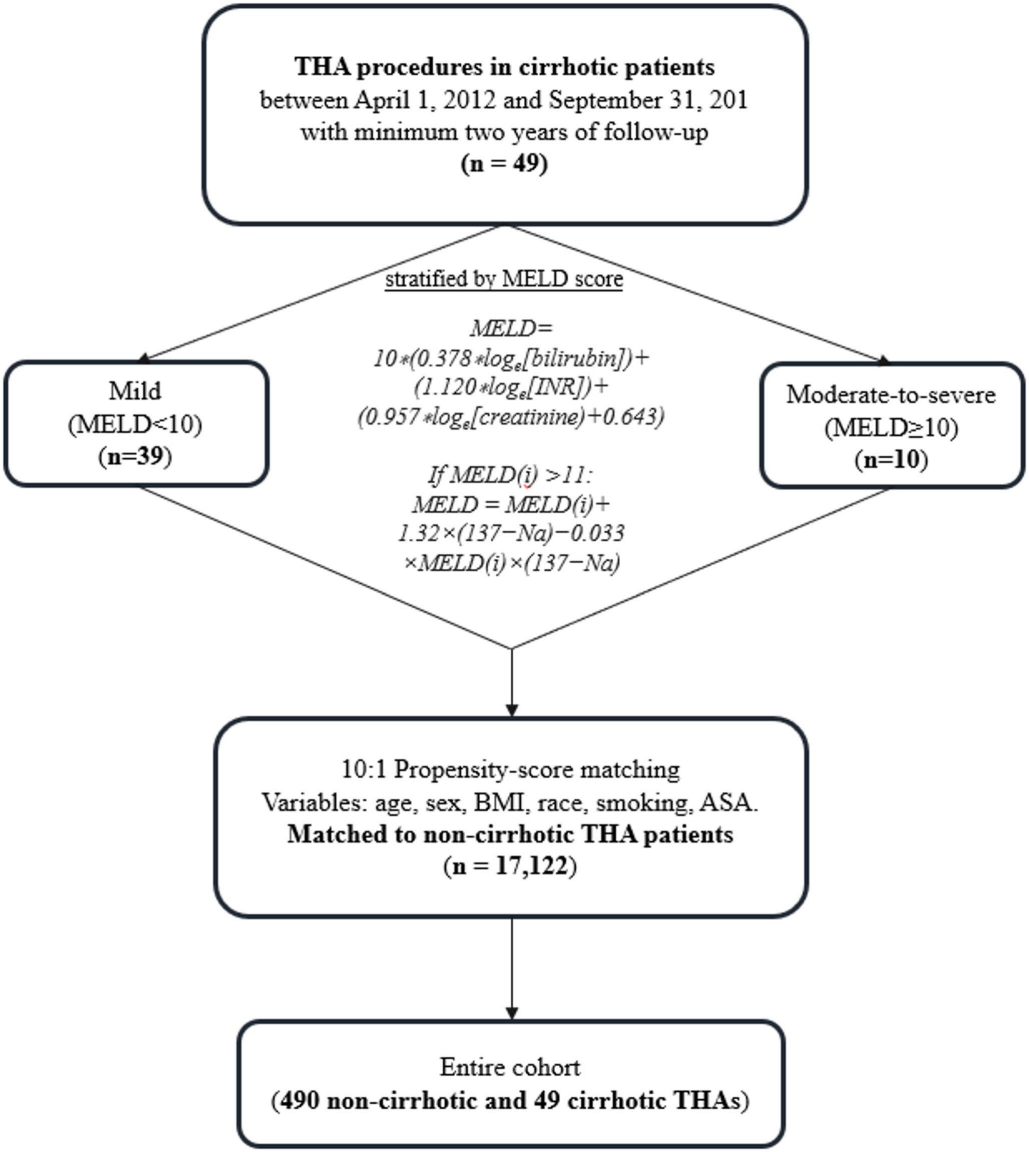
Flow diagram demonstrating patient selection and matching of non-cirrhotic and cirrhotic THA patients. THA, Total hip arthroplasty; n, Number; MELD, Model for end-stage liver disease; BMI, Body-mass index; ASA, American Society of Anesthesiologists

The MELD score incorporates four routinely measured laboratory parameters — creatinine (mg/dL), bilirubin (mg/dL), international normalized ratio (INR), and sodium (Na) (mEq/L)—along with a modifier reflecting recent dialysis (defined as at least two sessions in the previous week or continuous veno-venous hemodialysis for ≥ 24 h) [10]. The initial MELD score is calculated using the formula: 10 × (0.378 × log_e_[bilirubin] + 1.120 × log_e_[INR] + 0.957 × log_e_[creatinine] + 0.643). If this initial score exceeds 11, an additional adjustment accounts for serum sodium: MELD = MELD_(i)_ + 1.32 × (137 − Na) − 0.033 × MELD_(i)_ × (137 − Na). For patients who underwent dialysis at least twice in the preceding week, the serum creatinine value is set to 4.0. The MELD scores range from a minimum of 6 to a maximum of 40. Using chart review, components of the MELD score were extracted, using the most recent preoperative laboratory values obtained within 14 days before surgery. After calculating the MELD score, patients were stratified based on their MELD score as mild (MELD < 10, *n* = 39) or moderate-to-severe (MELD ≥ 10, *n* = 10). This stratification threshold was selected based on a prior study [11] that identified MELD ≥ 10 as a clinically meaningful cutoff associated with increased perioperative risk in cirrhotic patients undergoing non-transplant surgery (Fig. 1).

Clinical outcomes were collected via retrospective chart review including length of hospital stay (LOS), discharge disposition, 30-day readmissions, 90-day readmissions, 90-day septic and all-cause reoperations/revisions, and anytime reoperations/revisions until final follow up. The operative time was also collected for all procedures.

### Data analyses

Baseline patient characteristics were represented as counts with percentages for categorical variables and means with standard deviations for continuous variables. Other than descriptive statistics, the model consisted of *chi*-squared (x^2^) tests for assessing the differences in terms of categorical outcomes (sex, race, smoking status, ASA score, discharge disposition, 30-day and 90-day readmissions, 90-day and all-cause reoperations/revisions), and independent sample *t*-test to evaluate the differences of the means of continuous variables (age, BMI, CCI, operative time, LOS) between the cirrhotic and non-cirrhotic THA patients. Significance was defined as a *p*-value less than 0.05. In cases where a significant *p*-value was calculated, Cramer’s V (for categorical parameters) and Cohen’s d (for continuous parameters) were reported to demonstrate the effect size of the correlation: ≤ 0.2 (weak association), 0.2–0.6 (moderate association), ≥ 0.6 (strong association). Kaplan-Meier survivorship curves were utilized to analyze implant survival from all cause revisions between cirrhotic and non-cirrhotic cohorts. Data was organized and stored using Microsoft Excel software (Microsoft Corporation, Richmond, WA). Propensity score matching was performed using R Statistical Software (Version 4.1.2; R Core Team, Vienna, Austria). *Chi*-squared (x^2^) tests, independent samples *t*-tests, and Kaplan-Meier analyses were conducted using SPSS Statistics (Version 28; IBM Corporation, Armonk, New York, USA).

## Results

### Patient demographics

Of the 49 THAs in patients with cirrhosis, 25 (51.0%) were in women and 24 (49.0%) were in men. Mean patient age at the time of operation was 61.2 (SD = 11.0) years. Mean patient BMI at the time of operation was 28.8 (SD = 5.2) kg/m^2^. The majority of patients were white (73.5%) and former smokers (57.1%). The most common ASA score was 3.0 (71.4%) and the mean CCI was 5.1 (SD = 2.4). The average MELD score for THA patients with cirrhosis was 8.4 (SD = 2.9), ranging between 6 and 24 (Table 1).

**Table 1:** Demographics of matched cirrhotic and non-cirrhotic THA patients

Parameter	Non-cirrhotic (*n* = 490)	Cirrhotic (*n* = 49)	*p*-value	SMD
*Sex*, n (%)			0.999	0.00
Female	250 (51.0)	25 (51.0)		
Male	240 (49.0)	24 (49.0)		
*Age* ± SD, years	61.3 ± 15.5	61.2 ± 11.0	0.930	0.01
*Race*, n (%)			0.907	0.07
White	374 (76.3)	36 (73.5)		
African American or Black	33 (6.7)	3 (6.1)		
Asian	12 (2.4)	1 (2.0)		
Other	71 (14.5)	9 (18.4)		
*Smoking status*, n (%)			0.998	0.00
Never	161 (32.9)	16 (32.7)		
Former	278 (56.7)	28 (57.1)		
Current	51 (10.4)	5 (10.2)		
*ASA*, n (%)			0.400	0.24
I	6 (1.2)	0 (0.0)		
II	80 (16.3)	6 (12.2)		
III	292 (59.6)	35 (71.4)		
IV	112 (22.9)	8 (16.3)		
*BMI* ± SD, (kg/m^2)^	28.8 ± 6.4	28.8 ± 5.2	0.978	0.00
*Mean CCI* ± SD	4.9 ± 3.6	5.1 ± 2.4	0.540	0.07
*Mean MELD score* ± SD	–	8.4 ± 3.5	–	

*SD* standard deviation, *n* number, % Percentage, *BMI* body-mass index, *ASA* American society of Anesthesiologists, *CCI* Charlson comorbidity Index, *MELD* model for end-stage liver disease, *SMD* standardized mean difference

*Significance higher than 0.05

### Quality metrics and complications

THA operative times were similar between the non-cirrhotic, mild cirrhotic, and moderate-to-severe cirrhotic groups (99.0 vs. 99.8 vs. 116.3 min, *p* = 0.748). There was no difference in LOS between the non-cirrhotic, mild cirrhotic, and moderate-to-severe cirrhotic patients (3.0 vs. 3.3 vs. 3.0 days, *p* = 0.238). Discharge disposition was similar among the groups (*p* = 0.150), as most of the non-cirrhotic, mild cirrhotic, and moderate-to-severe cirrhotic patients were discharged home following surgery (80.6% vs. 66.7% vs. 70.0%).

Compared to the non-cirrhotic and mild cirrhotic, moderate-to-severe cirrhotic patients demonstrated a greater (effect size (ES) = 0.204) all-cause 30-day readmission rate (2.9% vs. 2.6% vs. 30.0%, *p* = 0.011) and a moderately (ES = 0.252) increased 30-day readmission rate for prosthetic joint infection (PJI) (0.6% vs. 2.6% vs. 20.0%, *p* = 0.007). A similar trend was observed in 90-day readmissions, as compared to the non-cirrhotic and mild cirrhotic patients, the moderate-to-severe cirrhotic cohort had a weakly (ES = 0.142) higher all-cause 90-day readmission rate (5.9% vs. 2.6% vs. 30.0%, *p* = 0.038), and specifically for PJI (1.2% vs. 2.6% vs. 20.0%, *p* = 0.024).

While there was no significant difference in the all-cause 90-day reoperation/revision rate between non-cirrhotic, mild cirrhotic, and moderate-to-severe cirrhotic cohort (3.1% vs. 2.6% vs. 20.0%, *p* = 0.115), the 90-day reoperation/revision rate for PJI was significantly (ES = 0.199) higher in the moderate-to-severe cirrhotic group (1.2% vs. 2.6% vs. 20.0%, *p* = 0.024). Similarly, there were no significant differences in the all-cause reoperation/revision rate at any time point (4.3% vs. 5.1% vs. 20.0%, *p* = 0.202), but the 90-day reoperation/revision rate for PJI was (ES = 0.187) greater in the moderate-to-severe cirrhotic group (1.4% vs. 5.1% vs. 20.0%, *p* = 0.016) (Table 2).

**Table 2:** Clinical outcomes and surgical data of matched cirrhotic and non-cirrhotic THA patients

Parameter	Cirrhotic THAs (*n* = 49)			*p*-value (ES)
	Non-cirrhotic (*n* = 490)	Mild cirrhotic (*n* = 39)	Moderate-to-severe cirrhotic (*n* = 10)	
*Operative time* ± SD, minutes	99.0 (74.0)	99.8 (26.5)	116.3 (34.1)	0.748
*LOS* ± SD, days	3.0 (2.5)	3.3 (1.8)	3.0 (2.5)	0.238
*Discharge disposition*, n (%)				0.150
Home	395 (80.6)	26 (66.7)	7 (70.0)	
Acute rehabilitation center	23 (4.7)	1 (2.6)	1 (10.0)	
Skilled nursing facility	72 (14.7)	12 (30.8)	2 (20.0)	
*30-day readmissions*, n (%)	14 (2.9)	1 (2.6)	3 (30.0)	0.011* (0.204)
SSI/cellulitis/drainage	3 (0.6)	0 (0.0)	0 (0.0)	0.751
PJI	3 (0.6)	1 (2.6)	2 (20.0)	0.007* (0.252)
DVT	1 (0.2)	0 (0.0)	0 (0.0)	0.909
Sepsis	1 (0.2)	0 (0.0)	0 (0.0)	0.909
Orthopedic-related	2 (0.4)	0 (0.0)	0 (0.0)	0.826
Non-orthopedic related	4 (0.8)	0 (0.0)	0 (0.0)	0.148
*90-day readmissions*, n (%)	29 (5.9)	1 (2.6)	3 (30.0)	0.038* (0.142)
SSI/cellulitis/drainage	6 (1.2)	0 (0.0)	0 (0.0)	0.563
PJI	6 (1.2)	1 (2.6)	2 (20.0)	0.024* (0.199)
DVT	2 (0.4)	0 (0.0)	0 (0.0)	0.826
Sepsis	2 (0.4)	0 (0.0)	0 (0.0)	0.826
Orthopedic-related	7 (1.4)	0 (0.0)	0 (0.0)	0.511
Non-orthopedic related	7 (1.4)	0 (0.0)	1 (10.0)	0.186
*90-day reoperations/revisions*, n (%)	15 (3.1)	1 (2.6)	2 (20.0)	0.115
PJI	6 (1.2)	1 (2.6)	2 (20.0)	0.024* (0.199)
Instability	1 (0.2)	0 (0.0)	0 (0.0)	0.909
Wound drainage	3 (0.6)	0 (0.0)	0 (0.0)	0.751
PPF	5 (1.0)	0 (0.0)	0 (0.0)	0.619
*All-time reoperations/revisions*, n (%)	21 (4.3)	2 (5.1)	2 (20.0)	0.202
PJI	7 (1.4)	2 (5.1)	2 (20.0)	0.016* (0.187)
Instability	3 (0.6)	0 (0.0)	0 (0.0)	0.751
Aseptic loosening	2 (0.4)	0 (0.0)	0 (0.0)	0.826
Wound drainage	3 (0.6)	0 (0.0)	0 (0.0)	0.751
Polyethylene wear	1 (0.2)	0 (0.0)	0 (0.0)	0.909
PPF	5 (1.0)	0 (0.0)	0 (0.0)	0.619

*n* number of patients, % Percentage, *SD* standard deviation, *LOS* length of stay, *SSI* superficial surgical infection, *PJI* periprosthetic joint infection, *DVT* deep vein thrombus, *PPF* periprosthetic fracture, *ES* effect size: weak (ES ≤ 0.2), moderate (0.2 < ES ≤ 0.6), and strong (ES > 0.6)

*Significance higher than 0.05

**Fig. 2:**
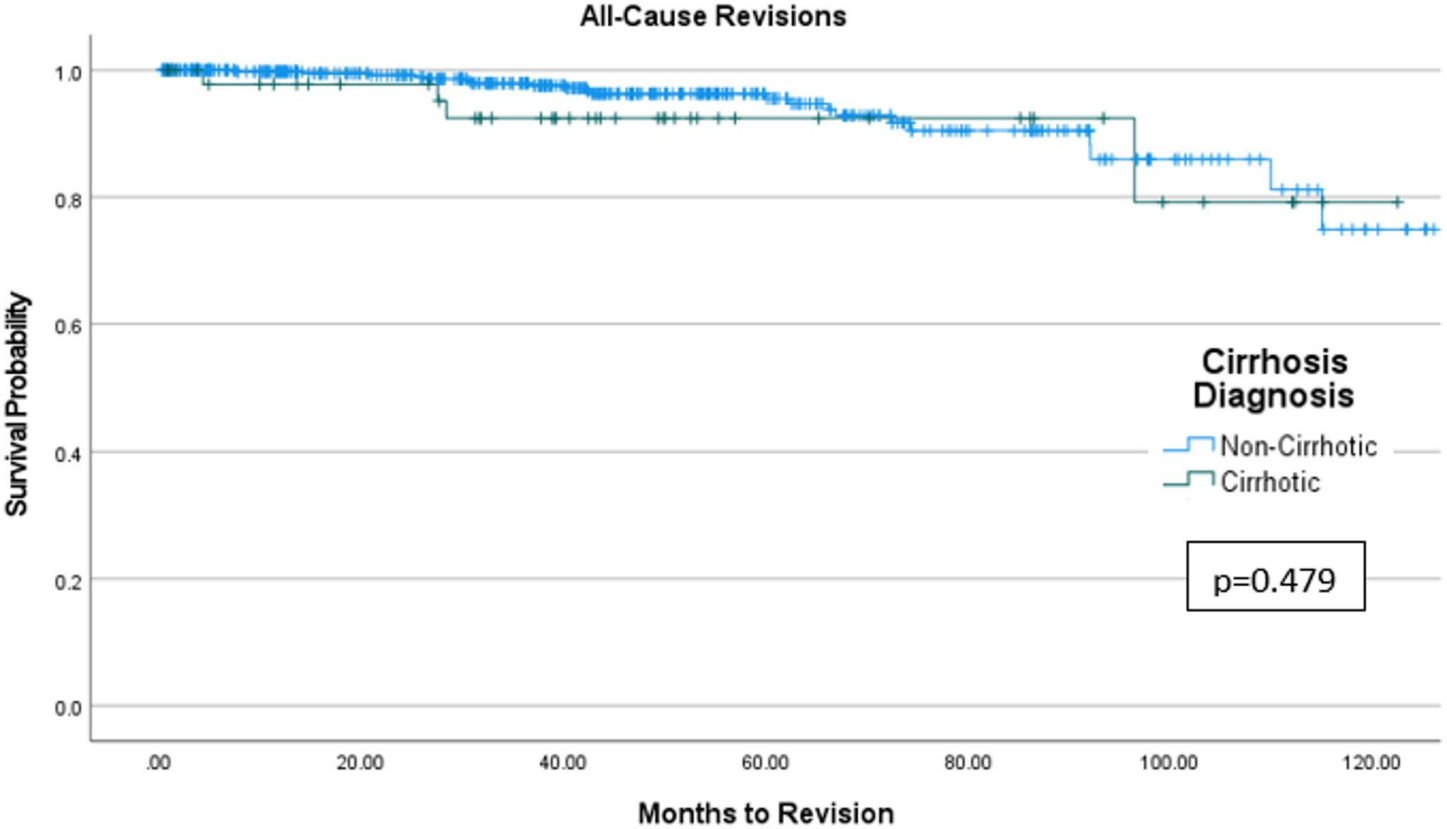
Kaplan-Meier Survivorship Curve demonstrating freedom from all-cause reoperations/revisions for both cirrhotic and non-cirrhotic total hip arthroplasty patients

Kaplan-Meier analysis showed similar implant survivorship between cirrhotic and non-cirrhotic THA patients with up to 120 months of follow-up (*p* = 0.479) (Fig. 2).

## Discussion

This study investigated the postoperative outcomes of THA in patients with and without cirrhosis, utilizing MELD scores to define and stratify the cirrhotic cohort into mild and moderate-to-severe groups. Our primary findings indicate that moderate-to-severe cirrhotic patients undergoing THA experience a significantly higher incidence of 30- and 90-day readmissions, and 90-day all-cause revisions particularly due to PJI, compared to non-cirrhotic THA patients. Furthermore, there were no differences in operative time, LOS, and discharge disposition between the investigated groups. Importantly, despite the increased risk for short-term postoperative complications, Kaplan-Meier analysis revealed similar long-term survivorship between non-cirrhotic and cirrhotic THA patients with up to ten years of follow-up.

Cirrhosis is a complex systemic disease that significantly impacts multiple physiological systems, thereby increasing the risk of adverse outcomes following surgical procedures, including THA [3, 12, 13]. Patients with cirrhosis often exhibit impaired immune function, coagulopathies, nutritional deficiencies, and hepatic encephalopathy, all of which can predispose them to postoperative complications [14]. Previous large database studies have consistently demonstrated an increased risk of complications and a higher financial burden in cirrhotic patients undergoing elective THA [4, 6]. Furthermore, several studies highlight that the increased comorbidity and complication rates in cirrhotic total hip and knee arthroplasty patients translate to significantly higher healthcare costs compared to their non-cirrhotic counterparts [4, 13, 15]. The average 8.5 MELD score in our cirrhotic cohort and the fact that only ten out of the 49 (20.4%) cirrhotic patients were moderate-to-severe cirrhotic patients suggest a population with predominantly mild liver disease, yet still susceptible to elevated risks and increased resource utilization. Despite this predominantly mild MELD score in our cohort, our findings still align with existing literature, underscoring that even mild cirrhosis presents a significant challenge in the context of THA. Future research should focus on developing prospective studies with larger cohorts of particularly moderate-to-severe cirrhotic patients, in order to further delineate the specific perioperative risks and outcomes associated with varying degrees of liver disease severity. This will inform surgeons and assist with preoperative risk stratification and discussion with cirrhotic patients considering THA.

In our cirrhotic cohort, particularly moderate-to-severe cirrhotic patients exhibited a significantly higher rate of 30- and 90-day readmissions, and a higher incidence of 90-day and all-cause revisions, predominantly driven by PJI. This observation is mainly clinically relevant given the devastating impact of PJI on patient outcomes and healthcare resources. The increased risk of PJI in cirrhotic THA patients is likely multifactorial, stemming from the aforementioned systemic immune dysfunction inherent to cirrhosis, which impairs the host’s ability to clear bacteria introduced during surgery or via hematogenous spread. Bacterial translocation from the gut, a common phenomenon in portal hypertension, may also contribute to the increased risk of deep space infection in a procedure like THA, which involves a relatively deep surgical field [16]. While previous studies [8, 9, 12, 17] have reported increased rates of infection and readmission in cirrhotic patients undergoing THA, it has been noted that the increased risk is rather an early and not a longer-term one. This aligns with findings from our study, as the significant complications in the cirrhotic THA cohort occurred only within 90 days of surgery (Table 2). Nevertheless, the 5.1% and 20.0% all-time infection rate observed in our mild and moderate-to-severe cirrhotic cohort, respectively, is lower than the previously reported 22.2% [17]. Furthermore, analysis demonstrated that there were no significant differences in all-cause reoperations/revisions between the groups. This suggests that while cirrhotic patients undergoing THA face an elevated risk of early complications, particularly PJI, the long-term need for additional surgeries may not be significantly different compared to non-cirrhotic patients. Future studies could delve deeper into the specific mechanisms of immune dysfunction and microbial translocation in cirrhotic patients undergoing THA, potentially identifying novel prophylactic strategies or targeted interventions to further reduce the risk of early PJI in this vulnerable population. Furthermore, given the relatively small number of cirrhotic patients in the present study, future investigation with large databases and or registries can help further solidify THA outcomes in cirrhotic patients. Additionally, larger, multi-center prospective studies with longer follow-up periods are warranted to confirm these findings and explore the nuances of long-term outcomes, including functional recovery and quality of life, in cirrhotic THA patients.

Operative time, LOS, and discharge disposition were not significantly different between the groups. These findings demonstrate that performing THA in cirrhotic patients, even those with mild or moderate-to-severe disease, does not inherently require an additional significant operative time. This is a critical observation, as prolonged operative time is often associated with increased risks of complications, including surgical site infections and blood loss [18, 19], yet our study found no significant difference in this metric between non-cirrhotic and cirrhotic groups. Furthermore, this similarity in operative parameters extends to LOS. Despite the underlying systematic vulnerabilities of cirrhotic patients, their average hospital stay post-THA was comparable to that of non-cirrhotic patients. As longer LOS often correlates with increased resource utilization, higher healthcare costs, and a greater risk of hospital-acquired complications such as nosocomial infections, and DVT, this finding suggests that the in-hospital management of cirrhotic THA patients, at least in terms of immediate postoperative recovery time, is not disproportionately burdensome, potentially reflecting effective perioperative care protocols [20–22]. Additionally, discharge disposition rates did not differ between the groups. This indicates that cirrhotic patient’s functional recovery and overall stability at the time of hospital discharge are sufficient for them to return to a home environment, rather than requiring transfer to a skilled nursing facility or long-term acute care hospital.

Despite the elevated short-term complications observed in cirrhotic THA patients, our Kaplan-Meier analysis showed no significant difference in freedom from all-cause reoperations/revisions for patients with cirrhosis compared to their non-cirrhotic counterparts. This long-term finding is clinically encouraging and suggests that while cirrhotic patients may encounter more immediate postoperative challenges, successful navigation of these early hurdles, perhaps through enhanced perioperative surveillance and management, can lead to comparable long-term implant survival. This finding contrasts with some studies [8, 23] that suggest a higher long-term revision burden in medically complex patients, although specific data on long-term revision rates in cirrhotic THA patients remain sparse. It implies that for carefully selected cirrhotic patients, THA remains a viable and durable option, provided meticulous perioperative management is implemented to address the heightened short-term risks, especially infectious complications.

### Limitations

This study, while providing valuable insights, is subject to several limitations that warrant consideration. The study’s retrospective, single-center design inherently introduces the potential for selection bias and confounding variables, despite the use of propensity score matching to minimize demographic differences between cohorts. The findings may also not be fully generalizable to other institutions with different patient populations, surgical volumes, or perioperative protocols. Furthermore, while a minimum of two years of clinical follow-up was required, a longer follow-up period could potentially reveal more late-occurring complications or differences in implant survivorship that were not captured within the study’s timeframe. Additionally, although the MELD score provides an objective measure of liver disease severity, it does not encompass the full spectrum of comorbidities or the fluctuating clinical status that can impact cirrhotic patients, potentially leading to residual confounding. Another limitation is the relatively small sample size of cirrhotic patients (*n* = 49) which might limit the statistical power to detect subtle yet clinically significant differences in outcomes for this subgroup. Finally, as with all studies relying on electronic medical record data, the accuracy and completeness of documented information, including diagnosis codes and complication reporting, are dependent on the fidelity of the clinical record.

## Conclusions

In conclusion, this study shows that moderate-to-severe cirrhosis significantly increases short-term complications after THA, particularly readmissions and revisions due to PJI. While operative time, LOS, and discharge disposition were similar across groups, emphasizing effective perioperative care, long-term implant survival up to ten years was comparable for both non-cirrhotic and cirrhotic patients. This suggests that despite early risks, THA can be a viable and durable option for carefully selected cirrhotic patients, provided meticulous attention is paid to mitigating immediate postoperative challenges, especially infection.

## Data Availability

No datasets were generated or analysed during the current study.
